# Interaction between a serotonin transporter gene promoter region polymorphism and stress predicts depressive symptoms in Chinese adolescents: a multi-wave longitudinal study

**DOI:** 10.1186/1471-244X-13-142

**Published:** 2013-05-17

**Authors:** Qing-sen Ming, Yun Zhang, Qiao-lian Chai, Hai-yan Chen, Chan-juan Hou, Meng-cheng Wang, Yu-ping Wang, Lin Cai, Xiong-zhao Zhu, Jin-yao Yi, Shu-qiao Yao

**Affiliations:** 1 Medical Psychological Institute, The Second Xiangya Hospital, Central South University, NO.139 Middle Renmin Road, Changsha, Hunan, 410011, P.R. China; 2 Hunan Province Technology, Institute of Psychiatry, Changsha, Hunan, 410011, P.R. China

**Keywords:** Depressive symptom, Serotonin transporter, Adolescent, China

## Abstract

**Background:**

The serotonin transporter (5-HTT) gene may play an important role in the onset and development of mental disorders. Past studies have tested whether a functional polymorphism in the 5-HTT gene linked promoter region (5-HTTLPR) moderated the association between stress and depressive symptoms, but the results of these studies were inconsistent. Thus, the aim of the current study was to examine the interaction between 5-HTTLPR and stress that predict depressive symptoms in Chinese adolescents.

**Methods:**

A total of 252 healthy adolescents (131 females and 121 males, aged from 14 to 18, mean = 16.00, standard deviation = 0.60) participated in this study. During the initial assessment, all participants completed the Center for Epidemiological Studies Depression Scale (CES-D) and Adolescent Life Events Questionnaire (ALEQ) and were genotyped for the 5-HTTLPR polymorphism. Participants subsequently completed CES-D and ALEQ once every three months during the subsequent 24 months. A multilevel model was used to investigate the 5-HTTLPR × stress interaction in predicting depressive symptoms.

**Results:**

The results indicated no main effect of 5-HTTLPR and a significant 5-HTTLPR × stress interaction in females only. Females with at least one 5-HTTLPR S allele exhibited more depressive symptoms under stressful situations. No significant 5-HTTLPR × stress interaction was found in males.

**Conclusions:**

In Chinese adolescents, there are gender differences on the interaction between 5-HTTLPR and stress that predict depressive symptoms. The association between stress and depressive symptoms is moderated by 5-HTTLPR in Chinese female adolescents.

## Background

Depression is a complex mental disorder, and its pathological mechanism has not yet been elucidated. A number of studies suggest that both genetic and environmental factors are involved in the onset or course of depression [1,2]. One environmental factor consistently linked to depression is exposure to stressful life events [3-6]. From the genetic viewpoint, the genes associated with the monoamine neurotransmitter serotonin (5-HT) have been well studied. 5-HT is an important neurotransmitter involved in regulating a number of psychological activities, such as emotion, cognition, and circadian rhythms. Functional impairment of the 5-HT system plays an important role in the pathogenesis of depression [7,8]. Among the genes of the 5-HT system, the serotonin transporter (5-HTT) gene has captured the most attention because 5-HTT is involved in the reuptake of serotonin at brain synapses, and it is the target of one of the most effective types of antidepressant medications, the selective serotonin reuptake inhibitors (SSRIs) [8]. The most researched functional polymorphism of the 5-HTT gene to date is the serotonin transporter-linked polymorphic region (5-HTTLPR). This polymorphism has two common alleles, the long (L) and short (S) alleles, differing by a 44-base pair (bp) insertion/deletion [9]. The S allele is associated with a lower transcriptional efficiency of the 5-HTT gene promoter compared with the L allele [10].

Caspi et al. [11] demonstrated that an interaction between 5-HTTLPR and stressful life events (5-HTTLPR × Stress) predicts depression in adults. Individuals with one or two copies of the S allele exhibited more depressive symptoms and diagnosable depression in relation to stressful life events than individuals homozygous for the L allele. Since then, other studies have been carried out to test the 5-HTTLPR × Stress interaction among adults, but the results of these studies were inconsistent. Some studies demonstrated an interaction [12,13], but others failed to replicate these results [14,15]. A meta-analysis published by Karg et al. [16] in 2011 supported that 5-HTTLPR moderates the relationship between stress and depression.

The current study aims to address several major limitations in the past research. First, the studies conducted to date used mainly cross-sectional designs and between-subject analyses. Thus, most studies tested 5-HTTLPR, in conjunction with between-subject differences in levels of stress, on predicting individual differences in depression. The current study addresses this shortcoming by utilizing a multi-wave longitudinal design in which levels of stress and depressive symptoms are assessed at multiple time points during the course of the 24-month follow-up interval. We examined 5-HTTLPR, in conjunction with within-subject fluctuations in levels of stress, on predicting within-subject fluctuations in levels of depressive symptoms. More specifically, we examined whether the slopes of the relationship between negative life events and depressive symptoms within individuals varied across individuals with different 5-HTTLPR genotypes.

Second, most studies have portrayed depression as a qualitatively distinct disorder and have used the incidence and diagnoses of depressive disorder as outcome variables. Nevertheless, several researchers suggested that depression may best be viewed as a quantitative deviation from “normal” affective experience and that it is a dimensional, not a categorical, construct [17,18]. Therefore, the current study treats depression as a continuous variable and assesses it by self-report depression measures.

Third, the majority of former studies that tested the 5-HTTLPR × Stress interaction in predicting depression focused predominantly on adult samples. Depressive symptoms and diagnoses increase dramatically at the onset of adolescence, particularly for girls [19]. Longitudinal studies indicate that a gender difference in depression appears at approximately age 13–15 [20]. Thus, adolescence is a crucial time to study the pathogenesis of genetic and environmental factors on depression. Moreover, a few studies focusing on youth samples indicated that the 5-HTTLPR × Stress interaction effect was not robust, particularly among boys, and that it may be moderated by gender [21,22]. The current study expounds the 5-HTTLPR × Stress interaction effect and gender differences in adolescent samples.

In addition, there has been no related research about the 5-HTTLPR × Stress interaction on depression among Chinese adolescent samples. Due to ethnic differences, it is necessary to test whether the conclusions of studies among other races may apply to Chinese adolescents. The current study was the first to collect Chinese adolescent samples and apply a longitudinal design for examining stress, 5-HTTLPR, and the 5-HTTLPR × Stress interaction in predicting depressive symptoms.

## Methods

### Participants

The study sample consisted of 252 healthy students (131 females and 121 males) ranging from 14 to 18 years old (M = 16.0, SD = 0.6). All the participants were selected from a public senior high school in Hunan province in mainland China. Class-based samples from 6 classes were selected randomly from all 10 senior grade one classes. All of the subjects are Han, the predominant ethnic group in China.

### Procedure

The study was approved by the Ethics Committee at the Second Xiangya Hospital, Central South University. All participants and their parents received detailed information and provided written consent. Trained researchers who were graduated students from the Second Xiangya Hospital administered both the clinical assessment and questionnaires to the subjects. Neurological physical examination and the interview of the Chinese version of the Schedule for Affective disorder and Schizophrenia for School-Age Children (K-SADS) [23] were conducted one-on-one with each participant outside of class time. Participants who had neurologic diseases, past or current episodes of major depression disorder, manic disorder, bipolar disorder, schizoaffective disorder and schizophrenia were excluded. At initial assessment, every participant completed the Chinese version of the following questionnaires: (1) Center for Epidemiological Studies Depression Scale (CES-D) [24], and (2) Adolescent Life Events Questionnaire (ALEQ) [25]. In addition, 5 ml of venous blood of each participant was collected in an EDTA vacuum tube during the initial assessment. The researchers returned to the school to meet with the participants every three months for the subsequent 24 months (i.e., at 3 months, 6 months, 9 months, 12 months, 15 months, 18 months, 21 months, and 24 months). At each of the 8 follow-ups, every participant completed the CES-D and ALEQ.

### Measurements

The procedures for developing the Chinese version of the CES-D and ALEQ questionnaires were described in detail previously [6].

#### Schedule for Affective disorder and Schizophrenia for School-Age Children (K-SADS)

The K-SADS is a semi-structured clinical interview based on DSM-IV (American Psychiatric Association, 1994) criteria that assesses depressive disorders and schizophrenia in children [23]. The K-SADS has been shown to yield reliable diagnoses of depressive disorders and is frequently used in research on clinical child psychology [23]. In the current study, diagnosticians from the Second Xiangya Hospital were trained to criterion to obtain reliable clinical diagnoses based on DSM-IV criteria. The training program consisted of didactic instruction, conducting practice interviews, and passing a diagnostic exam with an expected minimum score of 85%. The primary investigator of this study held weekly supervision sessions with the diagnosticians and reviewed interviewers' notes and tapes in order to obtain reliable diagnoses. Discrepancies were resolved through consensus meetings and best estimate procedures. Additionally, inter-rater reliability of the K-SADS was tested on diagnosis of 3 students. Nine raters were randomly selected from 21 raters. The Fleiss kappa was 0.73.

#### Center for Epidemiological Studies Depression Scale (CES-D)

Radloff [24] compiled the CES-D, which is a 20-item measure designed to assess the current level of depressive symptoms, with emphasis on the affective component, and depressive mood in general populations. Each item consists of one symptom. Participants rated the frequency of each symptom within the past week on a four-point scale of 0 (less than 1 day), 1 (1–2 days), 2 (3–4 days), or 3 (5–7 days). The total scores ranged from 0 to 60, with higher scores indicating higher elevations in depressive symptoms. The Chinese version of CES-D shows a high degree of reliability and validity [26]. The Cronbach’s alphas ranged from 0.89 to 0.96 across administrations, indicating strong internal consistency.

#### Adolescent Life Events Questionnaire (ALEQ)

The ALEQ is a self-report questionnaire that was developed by Hankin and Abramson [25] to assess a broad range of negative life events (e.g., school/achievement problems, friendship and romantic problems, and family problems) that occur during adolescence. The participants rated the frequency of negative life events within the past month on a five-point scale of 1 (never), 2 (rarely), 3 (sometimes), 4 (usually), or 5 (always). Total scores ranged from 70 to 350, with higher scores reflecting a greater number of negative life events. Past research found that the ALEQ is both reliable and valid [25]. In the current study, the Cronbach’s alphas ranged from 0.95 to 0.97 across administrations, indicating high internal consistency.

### 5-HTTLPR genotyping

Genomic DNA was extracted from the venous blood samples using the TIANamp Blood DNA Kit (TIANGEN Biotech, China) according to standard procedures. The genotyping of the 5-HTTLPR polymorphism was performed using the primers described by Heils et al. [9] (forward: 5′-GGCGTTGCCGCTCTGAATTGC-3′; reverse: 5′-GAGGGACTGAGCTGGACAACCCAC-3′). Polymerase chain reaction (PCR) amplification was conducted in a Perkin-Elmer GeneAmp PCR System 2400 (Applied Biosystems, USA). The amplification system was in a volume of 25 μL containing 50 ng of DNA template, 9.5 μL of nuclease-free water, 0.4 μM of each primer, 0.2 mM dNTPs, 10 mM Tris–HCl (pH = 8.3), 50 mM KCl, 1.5 mM MgCl_2_, and 1.25 U of GoTaq DNA polymerase (Promega, USA). The cycling conditions were as follows: (1) initial denaturation at 94°C for 3 min; (2) 35 cycles of amplification: denaturation at 95°C for 30 s, annealing at 62°C for 30 s, and synthesis at 72°C for 45 s; and (3) final extension at 72°C for 7 min. The amplification products were resolved on a 1.5% agarose gel by electrophoresis and visualized by Du Red staining (Biosharp, USA). Fragment sizes were determined on a Bio-Rad Gel Doc XR + system (Bio-Rad, USA) by comparison with molecular length standards (50 bp ladder, TIANGEN Biotech, China).

### Statistical analysis

Multilevel modeling was used to investigate whether the interaction between 5-HTTLPR and stress can predict the level of depressive symptoms. Analyses were carried out using the SAS (version 9.0) MIXED procedure and maximum likelihood estimation. The dependent variables were within-subject fluctuations in the CES-D scores during the follow-up interval (DEPRESSION). The primary predictors of DEPRESSION were 5-HTTLPR and fluctuations in the ALEQ scores during the follow-up interval (STRESS). As STRESS was a within-subject predictor, the ALEQ scores were centered at each participant’s mean prior to the analyses, such that STRESS reflects the upwards or downwards fluctuations in a participant’s level of stress compared to his/her mean level of stress. Preliminary analyses indicated that the interaction between 5-HTTLPR and STRESS was moderated by gender. Thus, the analyses were presented for the sample in two parts (i.e., for males and females).

## Results

### Frequency of 5-HTTLPR genotypes

The frequency distributions of the 5-HTTLPR genotypes are shown in Table 1. The genotype frequencies were consistent with Hardy-Weinberg equilibrium (*χ*^*2*^ = 0.045, *p* > 0.05) and were comparable to other studies in Chinese samples [27,28] (*p* > 0.05). No significant gender differences were found in the frequency distributions of 5-HTTLPR (*χ*^*2*^ = 0.19, *p* > 0.05).

**Table 1:** Genotype distributions of 5-HTTLPR

**Genotypes**	**Females**	**Males**	**Total**
LL	11(0.084)	12(0.099)	23(0.091)
SL	56(0.427)	50(0.413)	106(0.421)
SS	64(0.489)	59(0.488)	123(0.488)

### Descriptive DEPRESSION and STRESS data

At the initial assessment, the mean CES-D and ALEQ scores were 11.98 (SD = 8.72) and 116.68 (SD = 29.47), respectively. Pearson’s correlation coefficient between the CES-D and ALEQ scores was 0.43 (*p* < 0.001). More frequent negative life events were associated with higher levels of depression symptoms. There were no gender differences in either the CES-D scores or the ALEQ scores.

The means and standard deviations of all of the follow-up measures are presented in Table 2. There was an overall decrease in CES-D and ALEQ scores. Males reported higher levels of depression symptoms than females at follow-up 5 and follow-up 6 (*p* < 0.05). Females reported more frequent negative life events than males at follow-up 1 (*p* < 0.05), but less frequent negative life events than males at follow-up 3 and follow-up 5 (*p* < 0.05). However, there were no gender differences at the other follow-up assessments in depression symptoms and negative life events (Table 2).

**Table 2:** Means and standard deviations for all follow-up measures

	**Females**	**Males**	**Total**
**DEPRESSION**			
Follow-up 1	10.50 (9.54)	11.69 (9.42)	11.07 (9.48)
Follow-up 2	8.87 (8.84)	8.86 (8.64)	8.86 (8.73)
Follow-up 3	9.61 (9.45)	10.24 (11.03)	9.91 (10.23)
Follow-up 4	7.28 (8.71)	7.89 (9.33)	7.57 (9.00)
Follow-up 5	8.62_a_(8.98)	9.50_b_(10.46)	9.04 (9.71)
Follow-up 6	7.09_a_(7.97)	8.67_b_(10.31)	7.85 (9.19)
Follow-up 7	6.85 (8.54)	7.95 (9.57)	7.40 (9.07)
Follow-up 8	7.05 (9.18)	7.96 (9.58)	7.49 (9.37)
**STRESS**			
Follow-up 1	95.12_a_(24.60)	92.04_b_(29.75)	93.67 (27.14)
Follow-up 2	92.57 (25.33)	90.21 (28.44)	91.50 (26.76)
Follow-up 3	85.59_a_(21.75)	91.85_b_(31.96)	88.58 (27.23)
Follow-up 4	83.09 (22.24)	85.45 (27.61)	84.20 (24.89)
Follow-up 5	84.12_a_(23.70)	90.68_b_(32.01)	87.25 (28.10)
Follow-up 6	83.42 (23.40)	84.36 (26.62)	83.87 (24.95)
Follow-up 7	81.68 (22.07)	80.37 (25.97)	81.02 (24.05)
Follow-up 8	80.55 (23.30)	78.83 (24.11)	79.70 (23.67)

Note: Means with different subscripts significantly differ (*p* < 0.05).

### Statistical analyses of interaction between 5-HTTLPR and STRESS

To estimate the effects of 5-HTTLPR and STRESS on DEPRESSION during the follow-up intervals, a multilevel model was constructed. 5-HTTLPR was treated as a three-classification variable by triallelic genotyping. To control for individual differences in baseline depressive symptoms, the initial assessment (Time 1) CES-D scores were included in this model. The two-level models for subject *i* at Time *t* are as follows:

Level 1 (within-subject)

$${\text{DEPRESSION}}_{\mathrm{ti}}=\beta_{0i}+\beta_{1i}{\left(\text{STRESS}\right)}_{\mathrm{ti}}+e_{\mathrm{ti}}$$

Level 2 (between-subject)

$$\begin{array}{l} \beta_{0i}=\gamma_{00}+\gamma_{01}{\left(\text{Time}\;1\;\mathrm{CES}-D\right)}_{i}\\ \;+\gamma_{02}{\left(5-\mathrm{HTTLPR}\right)}_{i}+u_{0i} \end{array}$$

$$\beta_{1i}=\gamma_{10}+\gamma_{11}{\left(5-\mathrm{HTTLPR}\right)}_{i}+u_{1i}.$$

**Figure 1:**
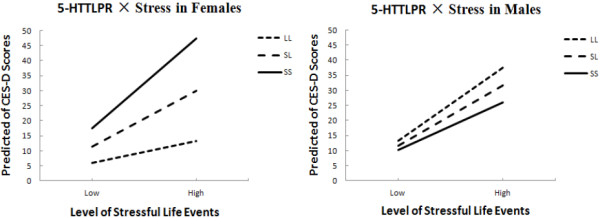
**The predicted slope between stress and depressive symptom for different genders and genotypes.** The high/low levels of stressful life events meant 1.5 within-subject standard deviation above/below individual’s mean level of stress. Predicted scores of CES-D were calculated by high/low stress scores using HLM. Females with one or two S alleles had significantly higher CES-D scores with high level of life events (**left panel**); but there were no association between life events and 5-HTTLPR predicting depressive symptoms for males (**right panel**).

The sample was divided into two parts (females and males). The fixed-effects components of the model were estimated in females and males, respectively. As shown in Table 3, the main effects of STRESS were significant both in females (*β* = 0.13, *p* < 0.0001) and males (*β* = 0.14, *p* < 0.0001). No significant main effect of 5-HTTLPR was found either in females (*β* = 1.16, *p* > 0.05) or in males (*β* = 0.21, *p* > 0.05). Among females, a significant two-way, cross-level interaction between 5-HTTLPR and STRESS emerged (*β* = 0.08, *p* < 0.0001); however, in males, no significant interaction appeared (*β* = −0.03, *p* > 0.05). The predicted slopes of the relationship between stress and depressive symptoms for females and males with the SS, SL, and LL genotypes are shown in Figure 1.

**Table 3:** Estimation of stress, 5-HTTLPR and 5-HTTLPR × stress predicting depressive symptoms

**Gender**	**Predictors**	***β***	**SE**	***t***
Females	1. Initial Depressive Symptoms	4.32	0.52	8.34^***^
	2. Stress	0.13	0.01	9.69^***^
	3. 5-HTTLPR	1.16	0.81	1.44
	4. 5-HTTLPR × Stress	0.08	0.02	4.53^***^
Males	1. Initial Depression Symptoms	3.69	0.60	6.10^***^
	2. Stress	0.14	0.02	8.91^***^
	3. 5-HTTLPR	0.21	0.90	0.23
	4. 5-HTTLPR × Stress	−0.03	0.02	−1.60

Note: Initial Depressive Symptoms as assessed by Time 1 CES-D; Stress as assessed by with-in subject fluctuations in ALEQ scores during the follow-up interval.

^***^p < 0.0001.

## Discussion

The current study showed that both stress and depressive symptoms were highest at the initial assessment (Time 1) and decreased during the follow-up interval. A possible reason for this result is that the students had just entered senior high school when the initial assessment was carried out. This major transition may have involved more competition and academic pressures, causing stress and depressive symptoms to have been at the highest levels. As the students acclimated to senior high school, their stress and depression levels may have decreased. In addition, there may have been a practice effect due to the repeated follow-up assessments. Consistent with former epidemiological studies in large samples of Chinese adolescents in a similar age bracket [29,30], the results of this study might indicate that there was no gender differences in depression in Chinese adolescents aged 14 to 18.

No evidence of a 5-HTTLPR main effect was found in either females or males in the current study, in accordance with the original research of Caspi et al. [11] and other studies in adolescent samples [22,31,32]. However, some studies in adolescents have reported a main effect of 5-HTTLPR (e.g. [33]). Inconsistent results have been reported in studies of other age brackets [34,35]. According to the results of the current study, we suggest that there may be no detectable effect of 5-HTTLPR on depressive symptoms of Chinese adolescents.

The main aim of the current study was to examine whether an interaction between 5-HTTLPR and stress predicts depressive symptoms in adolescents. Results showed that a significant interaction effect emerged in females, but not in males. Our results were mainly consistent with several former studies in adolescents [36-38]. These studies indicated that the interaction between 5-HTTLPR and stress should be less robust among male adolescents. It is possible that the interaction effects of genetic variation and stress on depression are moderated by gender, perhaps due to the influence of gender differences in hormone levels on gene expression, serotonergic function, and neural development. For example, estrogen can stimulate a significant increase in 5-HT_2A_ binding sites, and it acts as a 5-HT modulator. Estrogen not only increases the number of 5-HT_2A_ receptor binding sites, but it also increases 5-HT synthesis, uptake, and imipramine binding; it decreases 5-HT_1_ receptor binding sites and 5-HT transporter mRNA; and it increases the prolactin response to 5-HT agonists [39-42]. Another explanation is the gender × genotype interaction. In other words, genetic factors may have differential, or perhaps even opposite, effects on responses to stress. Research has shown that females are generally more reactive to the depressive effects of stress than males are, and such effects appear to be amplified by genetic factors [43]. Wüst et al. [44] confirmed this effect in a study of the association between 5-HTTLPR and hypothalamic-pituitary-adrenal axis regulation. They found that the 5-HTTLPR SS genotype was associated with increased cortisol awakening responses in females. But in males, subjects with the LL genotype exhibited enhanced awakening responses while the SS subjects showed the lowest awakening responses. Therefore, these results may partially explain the gender differences in the effects of 5-HTTLPR on the relationship between stress and depression. In addition, gender differences may in turn explain some of the inconsistencies in former research findings. Some former studies reported no interaction of stress and 5-HTTLPR (e.g., [34]), and one reason might be that these studies did not test for gender effects and had mixed gender differences in their studies. Therefore, more research is required to determine how these mechanisms might account for the different effects of 5-HTTLPR on depressive symptoms in males vs. females exposed to stress.

The present study benefited from several strengths including 2-year repeated assessments and a multi-wave design, which increased its statistical power. We adopted an idiographic approach and generated a relatively reliable estimate of each adolescent’s degree of stress reaction thereby minimizing the effects of individual differences in variables. Furthermore, we used a general community sample of adolescents, as opposed to a clinical sample with biases that reduce the generalizability, and we applied accurate statistical tests. In addition, to our knowledge, this is the first report on the interaction between 5-HTTLPR and stress predicting depressive symptoms in a longitudinal study in Chinese adolescents aged 14 to 18.

However, some limitations in the current study provide avenues for future research. First, perhaps the most important limitation was the sample size, which limited the number of participants of each gender and genotype. It is important to examine a larger sample and to replicate these results so that we can be more confident in the conclusions. Second, self-report measures were used to assess negative life events, which may not be as accurate as contextual stress interviews. Further research would benefit from using contextual stress interviews in a multi-wave design. Third, the present study only examined the gene × environment interaction in adolescents who were 14 to 18 years old. Thus, future research is necessary to test whether the findings in the present study can be generalized to younger adolescents or children. Finally, we analyzed one specific type of 5-HTTLPR allele variation. Several other sequence variations and single nucleotide polymorphisms (SNPs) in 5-HTT and other genes have been reported to be associated with depression [45]. Therefore, future studies should examine other gene × environment or gene × gene × environment interactions.

## Conclusions

In conclusion, the current study indicates that there are gender differences in the interaction between 5-HTTLPR and stress that predict depressive symptoms in Chinese adolescents. The association between stress and depressive symptoms is moderated by 5-HTTLPR in female adolescents only. In consideration of the limitation of the small sample size, the conclusions of the current study should be examined in a large sample in future research. If the results can be replicated, they will contribute to exploring genetic and environmental factors in pathological mechanism of depression, and providing definite research evidences for prevention and treatment of depression in Chinese adolescents.

## Competing interests

All authors declare that they have no competing interests.

## Authors’ contributions

Author YSQ, ZXZ and YJY designed the study and wrote the protocol. Author ZY, CQL, CHY and HCJ took part in follow-up assessments and data collecting. Author MQS, WMC, WYP and CL managed statistical analysis. Author MQS completed the literature searches and the first draft of the manuscript. All authors read and approved the final manuscript.

## Pre-publication history

The pre-publication history for this paper can be accessed here:

http://www.biomedcentral.com/1471-244X/13/142/prepub
